# High precision localization of pulmonary nodules on chest CT utilizing axial slice number labels

**DOI:** 10.1186/s12880-021-00594-4

**Published:** 2021-04-09

**Authors:** Yeshwant Reddy Chillakuru, Kyle Kranen, Vishnu Doppalapudi, Zhangyuan Xiong, Letian Fu, Aarash Heydari, Aditya Sheth, Youngho Seo, Thienkhai Vu, Jae Ho Sohn

**Affiliations:** 1 Department of Radiology and Biomedical Imaging, University of California San Francisco, 505 Parnassus Ave, San Francisco, CA 94143 USA; 2 George Washington University School of Medicine, 2300 I St NW, Washington, DC 20052 USA

**Keywords:** Lung nodule, Lung cancer, Nodule detection, Deep learning, Machine learning

## Abstract

**Background:**

Reidentification of prior nodules for temporal comparison is an important but time-consuming step in lung cancer screening. We develop and evaluate an automated nodule detector that utilizes the axial-slice number of nodules found in radiology reports to generate high precision nodule predictions.

**Methods:**

888 CTs from Lung Nodule Analysis were used to train a 2-dimensional (2D) object detection neural network. A pipeline of 2D object detection, 3D unsupervised clustering, false positive reduction, and axial-slice numbers were used to generate nodule candidates. 47 CTs from the National Lung Cancer Screening Trial (NLST) were used for model evaluation.

**Results:**

Our nodule detector achieved a precision of 0.962 at a recall of 0.573 on the NLST test set for any nodule. When adjusting for unintended nodule predictions, we achieved a precision of 0.931 at a recall 0.561, which corresponds to 0.06 false positives per CT. Error analysis revealed better detection of nodules with soft tissue attenuation compared to ground glass and undeterminable attenuation. Nodule margins, size, location, and patient demographics did not differ between correct and incorrect predictions.

**Conclusions:**

Utilization of axial-slice numbers from radiology reports allowed for development of a lung nodule detector with a low false positive rate compared to prior feature-engineering and machine learning approaches. This high precision nodule detector can reduce time spent on reidentification of prior nodules during lung cancer screening and can rapidly develop new institutional datasets to explore novel applications of computer vision in lung cancer imaging.

## Introduction

The National Lung Screening Trial (NLST) demonstrated that low-dose computed tomographic (CT) screening of high risk patients can result in a 20% reduction in mortality, leading to organizations to update their guidelines for lung cancer screening [1]. The U.S. Preventive Services Task Force recommends annual low-dose CT screening for patients with ≥ 30 pack year smoking history, and Fleischner Society Guidelines provide specific details for follow-up of incidental pulmonary nodules [2, 3]. As a result of these changes, Smieliauskas et al. projected an increase in CT scans and radiologist workload for lung cancer screening throughout the U.S., especially in low income regions with higher rates of smokers [4]. With over 8.6 million individuals eligible for low-dose lung cancer screening each year, 575 screens must be performed per lung cancer death avoided [5, 6]. Moreover, longer workdays and the associated fatigue have been shown to decrease radiologist diagnostic accuracy for pulmonary nodules [7].

The workflow for nodule detection and evaluation can be time consuming for a radiologist. In addition to identifying nodules on a new CT, radiologists must identify old nodules from prior scans and determine if there has been any temporal change. Despite having the nodule axial-slice number available in prior radiology reports, the process of identifying old nodules to cross-reference on the new CT is labor intensive. While advances in deep learning and computer-aided nodule detection have shown promise in nodule identification [8, 9], they do not focus on augmenting this critical aspect of the lung nodule screening workflow—locating previously identified nodules to observe changes over time by utilizing prior knowledge available in radiology reports (i.e. axial-slice location).

In this study, we utilize Lung Nodule Analysis 2016 (LUNA) to develop the deep learning model and NLST to evaluate a computer vision model to automatically identify lung nodules using the axial slice number to improve accuracy, thus helping reduce the workload required for manual reidentification of previously labeled nodules.

## Methods

### Data

**Fig. 1:**
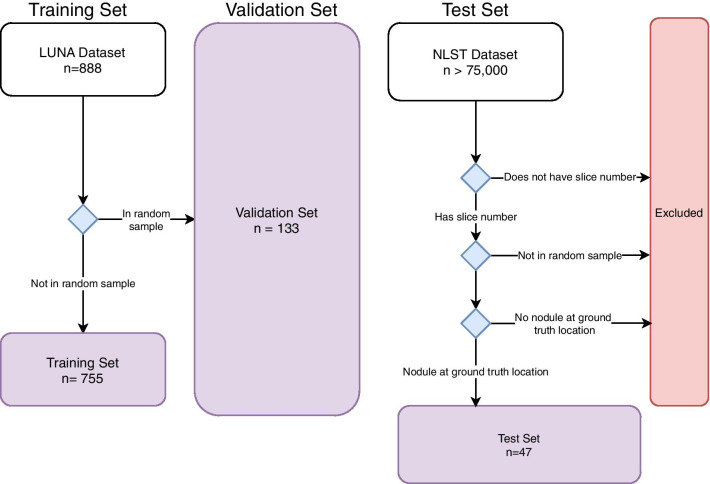
Cohort selection. Cohort selection for train/validation data and test data. Training data and test data are collected from two different sources. “n” refers to the number of CT scans. Each CT scan originates from a different patient

This study utilized the LUNA database for training the deep learning model and the NLST database for model evaluation. LUNA is a subset of the publicly available Lung Image Database Consortium (LIDC) dataset [10, 11]. LIDC contains 1018 anonymized helical chest CT scans positive for lung nodules and provides 3D coordinates for each nodule, which were determined by four thoracic radiologists. LUNA contains only CT scans from LIDC with a slice thickness of < 2.5 mm and classifies nodules with a diameter > 3 mm as “positive nodules.” LUNA data was split into a training (85%) and validation (15%) sets to optimize hyperparameters (Fig. 1). Prior to application on the external test set (NLST), the model was trained on the entire LUNA dataset to maximize performance by maximizing training set utilization.

NLST data, used for model evaluation, was accessed through the National Cancer Institute Cancer Data Access System with an approved Data Transfer Agreement. NLST data was anonymized prior to data transfer. NLST was conducted jointly by the NCI Division of Cancer Prevention’s Lung Screening Study (LSS) and the American College of Radiology Imaging Network. It consists of 53,454 patients enrolled between 2002 and 2004 in two study arms, chest X-ray and low-dose CT, to study the use of CT in lung cancer screening [1]. NLST provides annotations specifying the axial slice position of identified lung nodules, as well as diameter, lung region, and morphology. We randomly select 50 patients with 96 nodules from LSS sites from the CT-arm of the study to use in model evaluation (Fig. 1). 7 nodules were dropped because no nodule was identified at the ground truth axial-location by a chest radiology fellow. The final NLST test set includes 47 patients with 89 total nodules.

### Preprocessing

Axial CT scan pixel data was preprocessed to provide consistent real world scale regardless of originating CT dimensions or slice thickness. CTs were first transformed into 1 mm^3^ voxels, and then a 25 mm overlapping maximum intensity projection (MIP) was applied twice, once in the axial direction and again in the coronal direction. MIP allows for nodules to be easily distinguished from other lung features, especially blood vessels. However, blood vessels traveling perpendicular to the MIP can still appear as nodules. Therefore, we performed training and inference on both axial and coronal MIP projects. For training LUNA 3-dimensional nodule coordinates and diameters were used to create 2-dimensional bounding boxes for each axial and coronal MIP CT slice.

### Model

**Fig. 2:**
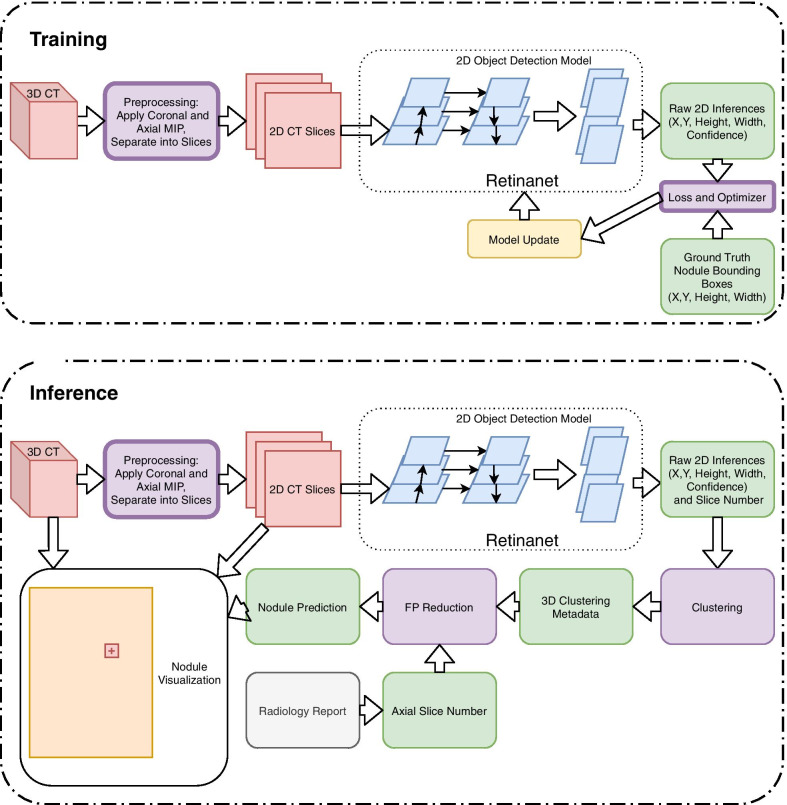
Algorithm schema. Training and inference pipeline for slice-assisted nodule detection. Training consists of using 2-dimensional axial and coronal MIP slices from each CT being input into a Retinanet model. Inference adds three additional steps to the Retinanet raw inferences: unsupervised clustering, false positive reduction using clustering metadata (max and mean confidence scores, whether the cluster contains both axial and coronal raw inferences, the number of raw inferences clustered together, and distance from top and bottom of the CT in mm)

While algorithm architecture contains multiple steps (Fig. 2), the base computer vision model is the 2-dimensional (2D) Retinanet, a state-of-the-art object detection algorithm [12]. We utilize an open-source implementation of Retinanet in PyTorch with resnet101 backbone pretrained on ImageNet [13, 14]. Focal loss, a modified cross-entropy loss that improves performance on object detection with extreme foreground:background imbalance (e.g. small nodules in a lung), was used with an Adam optimizer. A learning rate scheduler was used with an initial learning rate of 0.0005 and a reduction on plateau of validation set loss. A batch size of 2 was used. When training on the full LUNA data prior to inference on the NLST test set, no validation set was available. Therefore, the learning rate schedule was manually set to mimic prior model training with a validation set.

Model input consisted of 2D axial and coronal MIP slices. Data augmentation was randomly applied in real-time during training. Left–right and up–down flip was applied randomly to 50% of training slices in each batch. Slice height and width was independently scaled up to a 20% zoom. Slices were rotated up to ± 20 degrees, and shear was applied up ± 4 degrees. The Retinanet output consisted of bounding box coordinates and a confidence score for raw inferences for each input slice, and each slice may have more than one prediction.

### Post-processing

Post processing consists of two steps: aggregation of raw inferences into nodule candidates (Clustering) and final nodule predictions (False Positive Reducer and Axial-Slice Assisted Selection, Fig. 2).

#### Clustering

**Fig. 3:**
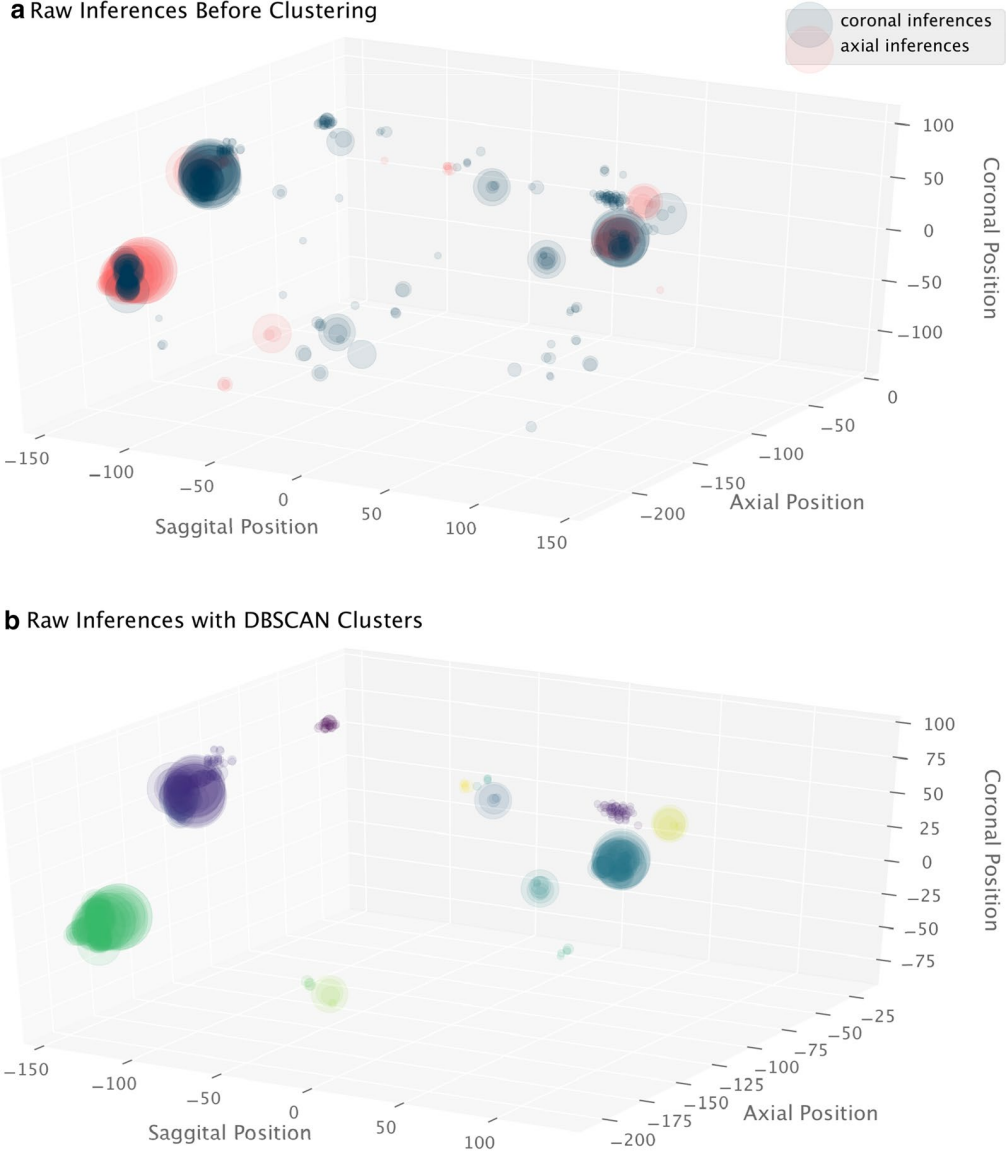
Unsupervised clustering of Retinanet raw inferences. Visualization of unsupervised clustering of raw Retinanet inferences using DBSCAN. Diameter of each production corresponds to the Retinanet inference confidence score. Higher density clusters containing both axial and coronal predictions with high confidence scores are more likely to be real nodules. The 3 large clusters in (**b**)—green, purple, and dark teal—were true nodules

Because raw inference predictions occur in a 2D plane, we utilized density-based spatial clustering of applications with noise (DBSCAN), an unsupervised density algorithm that identifies core high density regions and expands outwards to cluster raw inferences into discrete nodule candidates [15]. Prior to applying clustering, we filtered out any inferences with a confidence score of less than 0.1 to reduce background noise. DBSCAN required a minimum of 4 inferences to define a cluster, and the maximum distance for two inferences to be considered neighbors (eps) was set to 10 mm. The axial, coronal, and sagittal 3-dimensional position of inferences were used for clustering input (Fig. 3).

#### False positive reduction with cluster metadata

Clustering metadata was collected for use in false positive (FP) reduction (Fig. 2). This includes the max and mean confidence scores of clustered nodules, number of inferences belonging to each cluster, distance from top and bottom of CT scan, and whether a cluster contains both axial and coronal inferences. Clusters for true nodules were more likely to have higher confidence scores, more raw inferences, and both axial and coronal predictions. To utilize this metadata in FP reduction, we manually labeled a separate training set of 1380 clustered nodule predictions on 93 NLST CTs from 36 unique patients containing 165 true nodules—each patient could have up to 3 CTs from different years. It is important to note that none of these CTs or patients overlap with the NLST test set used for final evaluation of the nodule detector. We then trained XGBoost [16], a boosted tree classifier, on the aforementioned clustering metadata using GridSearch and fivefold cross-validation for hyperparameter search. A learning rate of 0.05, max tree depth of 6, 200 estimators, a scale positive weight of 2.7 to adjust for class imbalance, column subsampling of 0.6, and row subsampling of 0.8 were used in the final XGBoost classifier.

#### Axial-slice assisted selection

The final post-processing step was utilization of axial-slice labels from NLST. The FP Reducer was applied to the NLST test and would output a final nodule confidence score. Any nodules with a confidence score < 0.20 were automatically dropped as low-confidence FPs. Then, the closest remaining nodule within X distance of the specified axial slice number was selected as the final nodule candidate. X was tested at 10 mm and 20 mm.

### Evaluation

Predicted nodule candidates were visualized on the CT using Slicer3D [17]. Nodule predictions were evaluated by a chest radiology fellow and a medical student under the supervision of an attending chest radiologist. Precision, recall (sensitivity), and false positive rate per scan were calculated for distance thresholds of 10 mm and 20 mm and a confidence score of 0.20 (the minimum possible threshold) and 0.50. A free-response receiver operating characteristic curve was plotted at 10 mm and 20 mm distance thresholds. In seven instances, a true nodule was detected by our model but was different from the intended nodule, as determined by additional NLST data specifying lung lobe location and nodule visual features. To account for this, adjusted evaluation metrics were also reported with these cases marked as false positives. A manual error analysis was conducted of each incorrect and correct prediction to identify any patterns in errors with the detector. Demographic and nodule characteristics were compared between correct and incorrect/missed/unintended predictions using NLST metadata. Correct nodules were determined using a confidence score of 0.20 at a 20 mm threshold. Welch’s T-test and Chi-square test were used to compare continuous and categorical variables, respectively.

Model preprocessing, training, and testing code is available at https://bit.ly/3ivlFxt. Model was developed using Python 3.6 (Python Software Foundation).

## Results

### Demographics

The NLST test set consisted of 47 patients with a mean age of 62 years old that were mostly male and white (Table 1). 51% of patients were current smokers when undergoing their first NLST screening CT, and the remainder were former smokers. Patients had an average smoking history of 56.37 ± 34.33 pack years (Table 1). Among these 47 patients, there were 89 nodules, with an average incidence of 1.89 nodules per patient and an average diameter of 4.88 ± 2.26 mm per nodule (Table 2). Nodules characterized as soft tissue (88%) and smooth margins (82%) made up a majority of the nodules.

**Table 1:** NLST test set patient demographics

Variable	Missed/incorrect/unintended nodule predictions^a^ n (%)	Correct nodule predictions^a^ n (%)	*p*	Totals n (%)
Patients	15 (100%)	32 (100%)	–	47 (100%)
Age			0.586	
(mean ± SD)	61.07 ± 4.74	61.91 ± 5.12		61.64 ± 4.97
Sex			0.806	
Female	5 (33%)	8 (25%)		13 (28%)
Male	10 (67%)	24 (75%)		34 (68%)
Race			0.180	
White	12 (80%)	31 (97%)		43 (91%)
Black	1 (7%)	0 (0%)		1 (2%)
Asian	1 (7%)	1 (3%)		2 (4%)
> 1 Race	1 (7%)	0 (0%)		1 (2%)
BMI			0.256	
(mean ± SD)	29.72 ± 6.15	27.56 ± 5.35		28.25 ± 5.64
Smoker at start of NLST			0.468	
Yes	6 (40%)	18 (56%)		24 (51%)
No (former smoker)	9 (60%)	14 (44%)		23 (49%)
Cigarettes/day			0.251	
(mean ± SD)	32.67 ± 20.08	26.22 ± 9.00		28.28 ± 13.65
Smoking total years			0.978	
(mean ± SD)	39.87 ± 8.68	39.94 ± 7.03		39.76 ± 7.41
Smoking pack years			0.320	
(mean ± SD)	63.53 ± 54.91	51.62 ± 17.72		56.37 ± 34.33

Adjusted nodule performance with the highest recall score at a 20 mm distance threshold was used to split missed/incorrect/unintended and correct nodule predictions

*SD* standard deviation

^a^For patients with > 1 nodule, if at least one nodule was correctly identified for that patient, this patient was classified as a correct prediction

**Table 2:** NLST test set nodule characteristics

Nodule variable	Missed/incorrect/unintended nodule predictions n (%)	Correct nodule predictions n (%)	*p*	Totals n (%)
N	35 (100%)	54 (100%)	–	89 (100%)
Location			0.307	
Left lower lobe	7 (20%)	10 (19%)		17 (19%)
Left upper lobe	6 (17%)	7 (13%)		13 (15%)
Lingula	0 (0%)	6 (11%)		6 (7%)
Right lower lobe	12 (34%)	12 (22%)		24 (27%)
Right middle lobe	4 (11%)	10 (19%)		14 (16%)
Right upper lobe	6 (17%)	9 (17%)		15 (17%)
Central versus peripheral			0.332	
Central	3 (9%)	1 (2%)		4 (4%)
Peripheral	32 (91%)	53 (98%)		85 (96%)
Subpleural versus parenchymal			0.807	
Subpleural	19 (54%)	32 (59%)		38 (43%)
Parenchymal	16 (46%)	22 (41%)		51 (57%)
Margins			0.254	
Smooth	27 (77%)	46 (85%)		73 (82%)
Poorly defined	5 (14%)	5 (9%)		10 (11%)
Spiculated	1 (3%)	3 (6%)		4 (4%)
Unable to determine	2 (6%)	0 (0%)		3 (2%)
Diameter (mm)			0.752	
(mean ± SD)	4.77 ± 2.78	4.94 ± 1.75		4.88 ± 2.26
Attenuation			0.028*	
Soft tissue	27 (77%)	51 (94%)		78 (88%)
Ground glass	3 (9%)	1 (2%)		4 (4%)
Mixed	1 (3%)	2 (4%)		4 (4%)
Unable to determine	4 (11%)	0 (0%)		3 (3%)

Adjusted nodule performance with the highest recall score at a 20 mm distance threshold was used to split missed/incorrect/unintended and correct nodule predictions

*SD* standard deviation

*p < 0.05 using Chi-square test for categorical and Welch’s T-test for continuous variables to test for difference between correct and missed/incorrect nodule predictions

### Model results

**Fig. 4:**
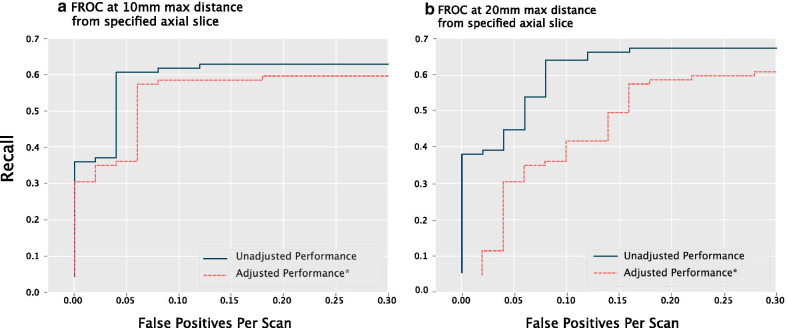
Free-response receiver operating characteristic at 10 mm and 20 mm thresholds. Low false positive (FP) rates were observed with axial-slice assisted selection of nodules. * adjusted to count only predictions on intended nodule as a true positive (e.g. if a calcified nodule was predicted, but the ground truth NLST label specified a ground glass nodule, this was recorded as an incorrect prediction)

At a confidence threshold of 0.50, our axial-slice assisted nodule detector was found to have a precision of 0.962 with a recall of 0.573 for identifying nodules at a 10 mm distance threshold and to have a precision of 0.931 and recall of 0.607 at a 20 mm distance threshold (Table 3). This translates to a false positive rate of 0.040 FP/scan (i.e. 1 FP every 25 scans) and 0.080 FP/scan (i.e. 1 FP every 12.5 scans) for 10 mm and 20 mm thresholds, respectively. When adjusting for cases where a different nodule was detected instead of the intended nodule, precision and recall both fall slightly to 0.943 and 0.561 at the 10 mm threshold and to the 0.862 and 0.562 at the 20 mm threshold. When utilizing a more sensitive confidence threshold (0.20), recall increased but precision fell slightly (Table 3). Figure 4, a free-response receiver operating characteristic (FROC) curve, visualizes recall at various low FP rates of < 0.30 FPs/scan. On the FROC curve, recall increases as the FP rate increases. At the 10 mm threshold, recall plateaus at a max of 0.629 (adjusted recall of 0.596), while the FP rate is zero at a recall of 0.360 (adjusted recall of 0.303). At the 20 mm threshold, recall plateaus at 0.674 (adjusted recall of 0.607), while the FP rate is zero at a recall of 0.382 (Fig. 4).

**Table 3:** Nodule detector performance

Performance metric	10 mm distance threshold	20 mm distance threshold
Nodule confidence score ≥ 0.50		
Precision	0.962	0.931
Recall	0.573	0.607
FPs/scan	0.040	0.080
Adjusted precision^a^	0.943	0.862
Adjusted recall^a^	0.561	0.562
Adjusted FPs/scan^a^	0.060	0.160
Nodule confidence score ≥ 0.20		
Precision	0.889	0.870
Recall	0.629	0.674
FPs/scan	0.140	0.180
Adjusted precision^a^	0.841	0.783
Adjusted recall^a^	0.596	0.607
Adjusted FPs/scan^a^	0.200	0.300

^a^Adjusted precision/recall/FPs counts only predictions on intended nodule as a true positive (e.g. if a calcified nodule was predicted, but the ground truth NLST label specified a ground glass nodule, this was recorded as an incorrect prediction)

### Error analysis

We stratified characteristics of patients (Table 1) and nodules (Table 2) using the 0.20 confidence score threshold and a 20 mm distance threshold by incorrect and correct nodule predictions. We found no difference between the 2 cohorts in any patient characteristics. The only nodule characteristic that differed between incorrect nodule predictions and correct nodule predictions was predominant attenuation (*p* = 0.028, Table 2). Compared to incorrect predictions, correct predictions had higher rates of soft tissue attenuation nodules (94% vs 77%) and lower rates of ground glass (2% vs 9%) and undeterminable nodules (0% vs 11%, Table 2).

**Fig. 5:**
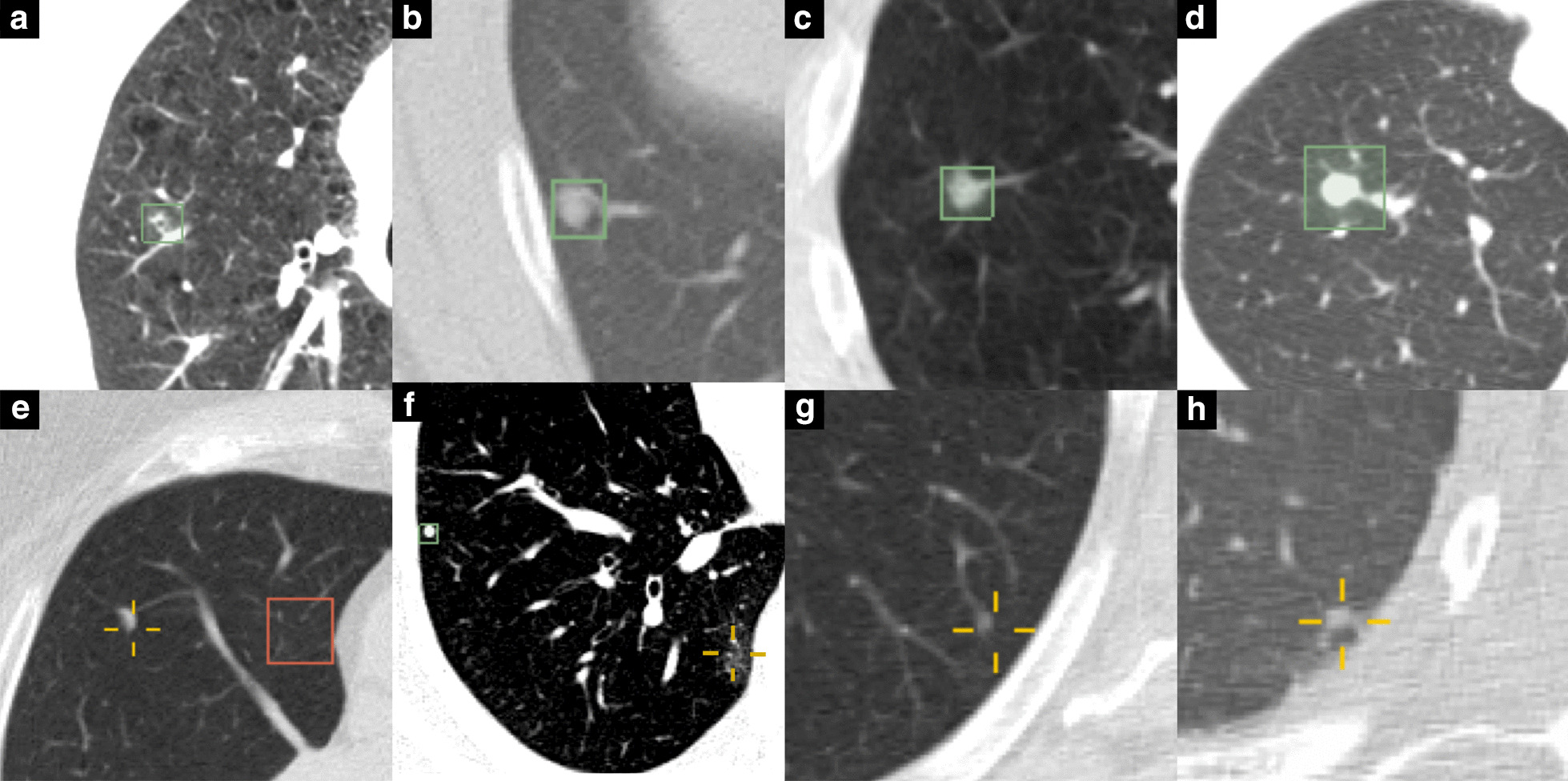
Prediction examples. Top row contains correctly identified nodules in green outline (**a**–**d**). Correctly identified nodules include ground glass nodule (**a**) and several soft tissue nodules (**b**–**d**). Bottom row contains missed nodules and false positives with yellow crosshairs specifying correct nodules (**e**, **f**). **e** FP prediction outlined in red. **f** Calcified nodule identified (green box) instead of ground glass nodule (yellow crosshair). **g** Small soft tissue nodule missed. **h** Small subpleural soft tissue nodule missed

Test set predictions were manually analyzed to identify trends in errors. Selected predictions are displayed in Fig. 5. Soft tissue (Fig. 5b–d) and calcified nodules (Fig. 5f) were more easily identified. The algorithm consistently struggled with ground glass nodules (Fig. 5f) and subpleural nodules (Fig. 5h). Due to the MIP and clustering step, the predicted location was occasionally slightly displaced from the true nodule position in areas with lots of “noise,” such as atelectasis. Notably, the average diameter of the predicted bounding box of correct nodules was 9.29 mm, nearly twice the value of the true diameters of these nodules (4.94 mm ± 1.75 mm, Table 2).

## Discussion

Re-identification of lung nodules on prior study is an essential task in lung cancer screening. We developed and evaluated a high precision end-to-end nodule detector that utilizes radiologist-defined axial-slice location, information which is widely available in radiology reports. Using a multi-step approach of 2D object detection with Retinanet, unsupervised clustering, and false positive reduction, our nodule detector can identify the coordinates of 57% of labeled nodules with a very low error rate of 1 FP every 25 scans (0.04 FPs/scan). Moreover, model testing was conducted on a different dataset (NLST, collected from 33 institutions) than training (LUNA, collected from 7 institutions), utilizing CT machines from several different companies (General Electric, Phillips, Siemens, and Toshiba), which suggests that this model is robust and generalizable to data from other institutions [1, 10].

In the past, a wide-array of techniques from feature-engineering approaches to deep learning techniques have been applied to nodule detection [18]. While feature-engineering techniques utilizing thresholding and edge detection struggled with FP/scans > 100, deep learning research has focused on optimizing recall at relatively lower FPs/scans values, ranging from 0.125 to 8 FPs/scan [11, 19, 20]. However, to the best of our knowledge, no prior approach has considered utilizing data commonly-found in radiology reports or emphasizing precision over recall. Axial-slice information is often reported as an image number (e.g. “3 mm pulmonary nodule in the right lower lobe—series 2, image 15—unchanged”). Existing CAD tools for nodule detection do not utilize this prior knowledge, which act as quasi-ground truth labels written by radiologists describing prior images.

In a theoretical clinical workflow, our nodule detector could extract 3D coordinates from axial-slice labels of prior CT images. Initially, these coordinates can be used to reduce search time for radiologists by simply visually highlighting nodules on prior CTs for easy comparison to new CTs. However, this model also lays the foundation for future research to utilize these 3D coordinates for a second nodule detection algorithm to focus on regions on the new CT that are near known nodule locations. For example, since the nodule should not shift significantly, a Gaussian kernel can be applied to the 3D coordinates to create a probability distribution that can be registered to lung areas in the new CT. This distribution can be utilized as an input for the second algorithm to predict nodules on the new CT. Additionally, once nodule coordinates are known for both old and new CTs, future computer vision research can focus on automatic analysis of the temporal change in nodule features to risk stratify patients. Since existing CAD systems do not utilize prior knowledge available in radiology reports, they are inherently limited in accuracy and ability to reliably analyze temporal change compared to models utilizing available information on prior nodule locations.

Unlike prior lung nodule detection methods, which often use 2-dimensional or 3-dimensional sliding windows to focus on high sensitivity, our pipeline inherently is geared towards high precision instead. By applying MIP, nodules can be easily visually distinguished from blood vessels. Furthermore, the dual-axis MIP (axial and coronal), allows nodules to be differentiated from blood vessels traveling perpendicular to one of the axes during the FP reduction step. Unsupervised clustering with DBSCAN has the advantageous property of considering spatial density when creating clusters, which reduces inclusion of nearby inferences that are not likely to be part of the nodule [15]. Moreover, clustering generates important metadata used in FP reduction. The most predictive features of a true nodule in the XGBoost FP reducer were a cluster having both axial and coronal inferences in a cluster and the total number of inferences clustered (feature importance scores of 0.659 and 0.157, respectively). This is intuitive as real nodules would be visible on both coronal and axial MIPs and would have many raw inferences on multiple slices due to MIP.

We foresee two potential applications of this high precision nodule detector: reduction of reading time for lung cancer screening and augmentation of research efforts applying deep learning to lung cancer screening. Observing changes in nodules over time is an important step in assessing malignancy risk of a nodule, and radiologists are required to re-identify nodules on CTs from prior time periods. While axial-slice location for prior CTs is annotated in radiology reports, the search processes for multiple nodules over many CTs may consume a considerable portion of a radiologist's time. Our nodule detector can automatically create X, Y, and Z coordinates to label nodules on prior CTs with high precision. Current computer-aided detection (CAD) systems struggle with relatively higher false positive rates, which limits their use in this setting. Christie et al. found that three commercial CAD systems achieved higher recall (0.82, 0.83, and 0.68) than our model at the expense of higher FP rates (0.62, 13.69, and 73.47 FPs/scan, respectively) on lung CT studies with an anthropomorphic thoracic phantom [21]. Notably, Christie et al. found that their two radiologists reference readers had FP rates of 0.14 and 0.27 nodules/scan, FP rates which are similar to those by our lung nodule detector [21]. For re-identification of labeled nodules, models with relatively higher FP rates may inadvertently lead to increased radiologist workload by having to evaluate each of the predictions manually. By focusing on a low FP rate, we believe that our nodule detector is more likely to reduce workload by reducing search time on prior reference-CTs.

**Fig. 6:**
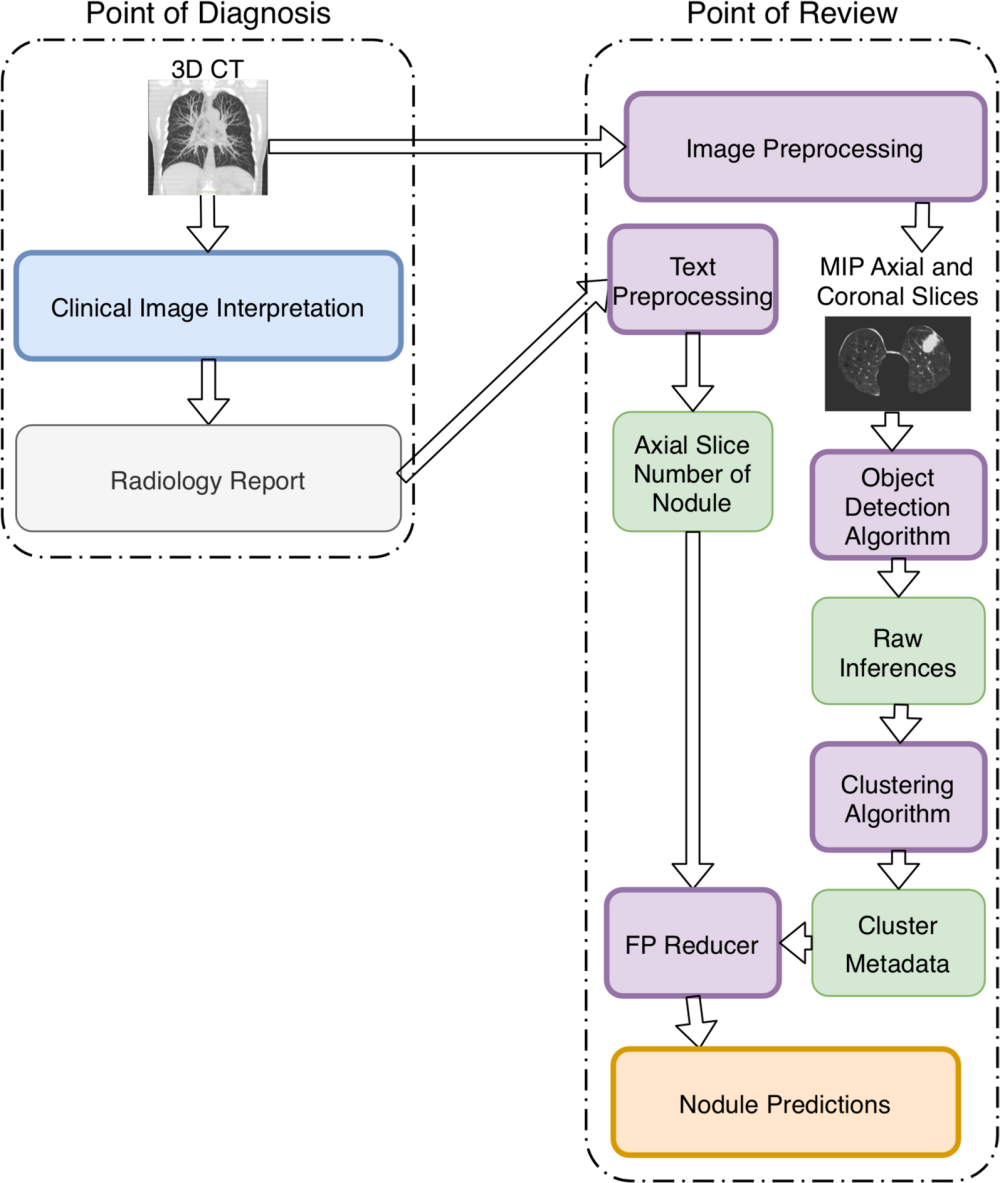
Clinical integration schema. Integration of the high precision nodule detector into clinical workflow. When reviewing a previously annotated lung cancer screening CT, the nodule detector will automatically highlight nodules using axial slice numbers derived from the radiology report

Clinical integration of deep learning models is a relatively new, but important, area of investigation and is warranted in future studies. New systems and methods have only recently been developed to trial these deep learning models in the clinic with existing hospital information technology infrastructure [22, 23]. We propose a basic schema for future clinical integration that utilizes text-preprocessing to extract axial-slice information, fully automating this pipeline (Fig. 6). In cases where our model failed to identify a nodule at an annotated axial-slice position, the corresponding slice would be marked on the PACS viewer to alert the radiologist of a missed nodule, further improving workflow efficiency. Existing solutions would allow this deep learning pipeline to run asynchronously, preventing disruption to the radiologist’s workflow [22]. Processing clinical free-text is another active area of research. However, we believe the increased standardization of lung cancer screening reports and advances in free-text processing, or more commonly natural language processing, will allow for accurate axial-slice extraction [24, 25]. Future integration research can aim to clarify these and other implementation details and quantify workload reduction.

In addition to the clinical impact, we believe a high precision detector can improve research efforts in applying deep learning techniques to lung cancer screening. There is a paucity of radiologic data with high quality labels for nodule detection, and lung nodules are no exception. One of the most popular datasets used to train and evaluate lung nodule detectors is LUNA, which contains only 888 CTs with 2290 nodules [11, 19, 20]. For comparison, the Common Objects in Context dataset is a benchmark dataset for everyday object detection tasks and contains over 200,000 images with over 1.5 million segmented objects [26]. A high precision end-to-end nodule detector allows researchers to utilize their own institutional data to rapidly build custom lung nodule datasets. These datasets can be applied to both improve nodule detection performance and also be used to address more complex problems. For example, future computer vision research can automatically characterize changes in nodules overtime to predict occurrence, type, and severity of cancer based on an initial lung nodule. To create these custom datasets with high-quality ground truth labels, a model must be developed with an emphasis on precision with reasonable recall.

Despite the high precision, we were able to identify consistent trends in missed nodules. Our classifier struggled to identify nodules with poorly defined margins or ground glass attenuation, which are associated with adenocarcinoma spectrum tumors. Computer vision models have consistently lower recall for non-solid nodule detection like ground glass opacities [11]. This may be partially due to a significantly lower rate of non-solid nodules available in the training set. Additionally, most focal ground glass opacities eventually turn out to be infection or inflammation. While LUNA does not contain nodule margin descriptions, only 7% of the NLST test set nodules were ground glass attenuation, suggesting a low general prevalence. As several past studies applying computer vision to nodule classification, rather than detection, have done, a dataset with high prevalence of ground glass nodules may have to be utilized to improve performance significantly [27, 28]. Additionally, it is important to note that in many cases, like Fig. 5f (yellow crosshairs), it is challenging if not impossible for radiologists to determine if a ground glass opacity is due to malignancy or other causes, such as inflammation or infection, with just a single time point. Temporal subtraction has been used to improve radiologist performance and could be integrated with deep learning-based nodule detectors in future studies [29].

Our study faces a few technical limitations. As we focused on building a high precision detector, our detector has a relatively low recall of nodules, which is inherent in the design and objective of the algorithm. This strength and weakness of the algorithm's accuracy profile should be taken into account in its clinical integration. Because MIP compresses information axially and coronally, this can lead to slight offsets in prediction. Similarly, when identifying nodule locations through clustering, taking the mean of the cluster can lead to an additional offset from the true nodule center. However, these offsets were slight, and the predicted nodules were well within bounding box diameters. Because we set the predicted bounding box diameter to the largest diameter of an individual 2D inference in the cluster, the bounding diameter values were nearly double the true nodule diameters. The bounding box diameter was not intended and should not be used as an estimate of the true nodule diameter.

## Conclusions

We developed a high precision axial-slice assisted lung nodule detector that can be utilized to improve radiology workflow during lung nodule screenings and augment research efforts in the application of deep learning to lung cancer detection. Future research can be directed at improving performance on ground glass nodule detection and utilize temporal lung nodule screening data to predict malignancy.

## Data Availability

The datasets supporting the conclusions of this article are available in the Grand Challenge: Lung Nodule Analysis 2016 repository (https://luna16.grand-challenge.org/download/) and the Cancer Data Access System: National Lung Screening Trial (Clinical Trial Number NCT00047385, https://cdas.cancer.gov/nlst/).

## Abbreviations

NLST: National Lung Screening Trial
CT: Computed tomography
LUNA: Lung Nodule Analysis 2016
LIDC: Lung Image Database
LSS: Lung screening study
MIP: Maximum intensity projection
DBSCAN: Density-based spatial clustering of applications with noise
FP: False positive
FROC: Free-response receiver operating characteristic
CAD: Computer-aided detection
2D: 2-Dimensional
